# Introduction to the Revised American Association for the Study of Liver Diseases Position Paper on Acute Liver Failure 2011

**DOI:** 10.1002/hep.25551

**Published:** 2012-03

**Authors:** William M Lee, R Todd Stravitz, Anne M Larson

**Affiliations:** University of Texas Southwestern Medical CenterDallas, TX

## Preamble

The present version of the American Association for the Study of Liver Diseases (AASLD) Position Paper represents a thorough overhaul from the previous version of 2005. In addition to two new additional authors, the revision includes updated expert opinion regarding (1) etiologies and diagnosis, (2) therapies and intensive care management, and (3) prognosis and transplantation. Because acute liver failure (ALF) is an orphan disease, large clinical trials are impossible and much of its management is based on clinical experience only. Nonetheless, there are certain issues that continue to recur in this setting as well as growing consensus (amidst innovation) regarding how to maximize the ALF patient's chance of recovery. The changes in ALF management are not global in nature, but are more consistent with incremental experience and improvements in diagnosis and intensive care unit management.

## Introduction

The diagnosis of ALF hinges on identifying that the patient has an acute insult and is encephalopathic. Imaging in recent years has suggested “cirrhosis,” but this is often an overcall by radiology, because a regenerating massively necrotic liver will give the same nodular profile as cirrhosis.^1^ It is vital to promptly get viral hepatitis serologies, including A-E as well as autoimmune serologies, because these often seem to be neglected at the initial presentation. Fulminant Wilson's disease can be diagnosed most effectively not by waiting for copper levels (too slow to obtain) or by obtaining ceruloplasmin levels (low in half of all ALF patients, regardless of etiology), but by simply looking for the more readily available bilirubin level (very high) and alkaline phosphatase (ALP; very low), such that the bilirubin/ALP ratio exceeds 2.0. 2 The availability of an assay that measures acetaminophen adducts has been used for several years as a research tool and has improved our clinical recognition of acetaminophen cases when the diagnosis is obscured by patient denial or encephalopathy. 3 Any patient with very high aminotransferases and low bilirubin on admission with ALF very likely has acetaminophen overdose, with the one possible exception being those patients who enter with ischemic injury. Obtaining autoantibodies should be routine and a low threshold for biopsy in patients with indeterminate ALF should be standard, given that autoimmune hepatitis may be the largest category of indeterminate, after unrecognized acetaminophen poisoning. 4

## Advances in Management of ALF

The medical management of ALF has not been extensively studied and remains poorly defined. In the absence of evidence-based clinical trials, experts from 23 centers in the United States have proposed detailed management guidelines by consensus.^5^ Since the last AASLD Position Paper, several noteworthy advances have been made in assessing the risk of developing, and managing, specific complications of ALF.

A detailed analysis of serum ammonia in patients with ALF identified a concentration of 75 μM as an important threshold below which patients rarely develop intracranial hypertension (ICH).^6^ Conversely, arterial ammonia levels of >100 μM on admission represent an independent risk factor for the development of high-grade hepatic encephalopathy, and a level of >200 μM predicts ICH. The risk of developing ICH is decreased by raising the serum sodium to 145-155 mEq/L with hypertonic saline. 7 Once established, however, the medical treatment of ICH must bridge patients to liver transplantation, because no treatment permanently reverses cerebral edema. In cases of ICH refractory to osmotic agents (e.g., mannitol and hypertonic saline), therapeutic hypothermia (cooling to a core temperature of 32°C-34°C) has been shown to bridge patients to transplantation, 8 but is associated with a theoretical risk of impairing liver regeneration.

To optimize neurological recovery after ALF, mean arterial pressure (MAP) and cerebral perfusion pressure (CPP) must be raised to avoid cerebral underperfusion and anoxia. In hypotensive patients with ALF, intravascular volume should be repleted first with normal saline, and vasopressors should be administered subsequently to titrate the MAP to >75 mmHg and CPP to 60-80 mmHg. Vasopressin, or its analog, terlipressin, is often added to norepinephrine in critically ill patients who remain hypotensive on norepinephrine, but was reported to increase intracranial pressure (ICP) in patients with ALF.^9^ More recent data suggest, however, that vasopressin and analogs increase cerebral perfusion without increasing ICP and may be used safely as an adjunct to norepinephrine. 10

It is generally accepted that patients with ALF have a bleeding diathesis based upon elevation of the international normalized ratio (INR). Concern about the safety of inserting ICP monitors and other invasive devices has prompted the use of recombinant factor VIIa,^11^ although the practice has been associated with thrombotic complications in patients with ALF. 12 However, a recent study has suggested that global hemostasis assessed by thromboelastography usually remains normal, suggesting that the perceived bleeding risk based upon INR may be overstated. 13

## Prognosis and Transplantation

To date, it often remains difficult to predict which ALF patients will ultimately require transplantation. Newer models, including the model for end-stage liver disease (MELD) score, have not improved our accuracy. In fact, the discriminative power of the MELD was not found to be superior to that of the INR or the King's College Hospital criteria.^14^ In addition, equating transplantation with death, in many models, inflates the positive predictive value of a particular system. The King's College Criteria remain the most clinically useful, with a sensitivity of 68%-69% and a specificity of 82%-92%. 15 However, reliance entirely upon any set of guidelines cannot be recommended.

Despite great early interest in liver support systems, the field has had little forward movement since our last publication. Both artificial (i.e., sorbent-based) and bioartificial (i.e., cell-based) systems have been tested. There has been no good evidence that any artificial support system reliably reduces mortality in the setting of ALF.^16^, ^17^ Thus, the currently available liver support systems cannot be recommended outside of clinical trials.

Liver transplantation remains the only definitive treatment for patients who fail to demonstrate recovery. The 1-year survival after cadaveric liver transplant for ALF is less than that observed in patients transplanted for chronic liver failure.^18^ However, after the first year, this trend had reversed and ALF patients have a better long-term survival. The use of live donor liver transplantation and auxiliary liver transplant remain controversial. 19 Urgent cadaveric liver transplantation remains the standard of care in the setting of ALF.

Developing effective methods of liver support or other alternatives to transplantation and better prognostic scoring systems remain key goals to further improve overall survival rates and avoid unnecessary transplants.

## Abbreviations

AASLD: the American Association for the Study of Liver Diseases
ALF: acute liver failure
ALP: alkaline phosphatase
CPP: cerebral perfusion pressure
ICH: intracranial hypertension
ICP: intracranial pressure
INR: international normalized ratio
MAP: mean arterial pressure
MELD: model for end-stage liver disease.
